# Neurodevelopmental care across pediatric critical care settings: toward brain-focused critical care

**DOI:** 10.1007/s00431-026-07203-y

**Published:** 2026-06-27

**Authors:** Sharon Morag, Tamar Bernstein, Melissa B. Jones, Gil Wernovsky, Uri Pollak

**Affiliations:** 1 Department of Neonatology, Dana Dwek Children’s Hospital, Tel Aviv Sourasky Medical Center, Tel Aviv, Israel; 2 Gray Faculty of Medical and Health Sciences, Tel Aviv University, Tel Aviv, Israel; 3 The Center for the Study of Child Development, The School of Psychological Sciences, University of Haifa, Haifa, Israel; 4 Division of Cardiac Critical Care, Children’s National Hospital, Washington, DC USA; 5 Pediatric Cardiology and Cardiac Critical Care, The George Washington University School of Medicine and Health Sciences, Washington, DC USA; 6 Section of Pediatric Critical Care, Hadassah University Medical Center, Ein Kerem, Jerusalem, Israel; 7 Faculty of Medicine, The Hebrew University of Jerusalem, Jerusalem, Israel

**Keywords:** Neurodevelopmental care, NICU, PICU

## Abstract

Advances in neonatal and pediatric critical care have expanded the focus from short‑term survival to include greater attention to long‑term neurodevelopmental health and family well‑being. This narrative review synthesizes the evolution of neurodevelopmental and neuroprotective care across neonatal (NICU), pediatric (PICU), and cardiovascular (CVICU/PCICU) intensive care settings. Developmental care emerged in the NICU through individualized, cue‑based approaches such as the Newborn Individualized Developmental Care and Assessment Program (NIDCAP), emphasizing stress reduction, protection of sleep, optimized sensory environments, and parent–infant coregulation. As survivorship after critical illness improved, parallel concerns about post‑intensive care morbidities, including cognitive, behavioral, and functional impairments, catalyzed adoption of family‑centered and brain‑focused practices in the PICU, supported by contemporary guidelines integrating pain and sedation optimization, delirium prevention, environmental stewardship, and early mobility. More recently, dedicated cardiac neurodevelopmental programs have adapted NICU principles to the high‑acuity CVICU/PCICU population, pairing hemodynamic vigilance with developmental goals through structured interdisciplinary models (e.g., developmental rounds, holding protocols, early therapy, feeding support, and caregiver mental health resources). Across settings, common implementation domains include family partnership, cue‑based care, protected sleep and circadian support, sensory modulation, humane pain and sedation strategies, early rehabilitation, and coordinated follow‑up after discharge.

*Conclusion*: While the strength of evidence varies by unit type and outcome, available data support feasibility and potential benefits for delirium reduction, functional recovery, feeding, parent experience, and early developmental trajectories. Continued multicenter research and implementation science are needed to define optimal bundles, equity‑informed delivery, and durable long‑term outcomes.

**Table:** 

**What is Known:** • *Neurodevelopmental care is well established in NICUs, emphasizing cue-based care, pain reduction, environmental protection, and family partnership.* • *Survivors of pediatric and cardiac critical illness remain at risk for cognitive, behavioral, emotional, and functional impairments.*
**What is New:** • *This review extends neurodevelopmental care beyond the NICU to PICU and CVICU settings.* • *It proposes a unified multidisciplinary framework for brain-focused critical care across pediatric ICU environments.*

**What is Known:**

• *Neurodevelopmental care is well established in NICUs, emphasizing cue-based care, pain reduction, environmental protection, and family partnership.*

• *Survivors of pediatric and cardiac critical illness remain at risk for cognitive, behavioral, emotional, and functional impairments.*

**What is New:**

• *This review extends neurodevelopmental care beyond the NICU to PICU and CVICU settings.*

• *It proposes a unified multidisciplinary framework for brain-focused critical care across pediatric ICU environments.*

## Introduction

**Fig. 1:**
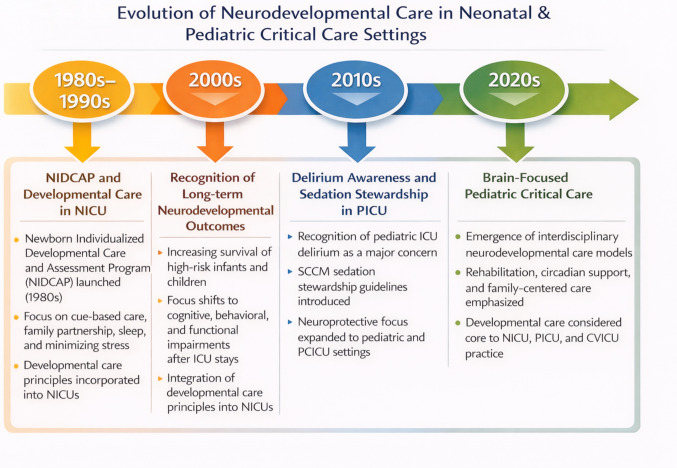
Evolution of neurodevelopmental care in neonatal and pediatric critical care settings. This timeline illustrates the progressive shift in neonatal and pediatric critical care from a survival-centered model toward a brain-focused, neurodevelopmentally informed approach. In the 1980 s and 1990 s, the Newborn Individualized Developmental Care and Assessment Program (NIDCAP) introduced individualized developmental care in the NICU, emphasizing cue-based care, family partnership, sleep protection, and stress minimization. During the 2000 s, improving survival of high-risk infants and children led to increasing recognition of long-term cognitive, behavioral, and functional outcomes after intensive care. In the 2010 s, pediatric critical care expanded its focus to include delirium awareness, sedation stewardship, and broader neuroprotective practices in PICU and PCICU settings. In the 2020 s, brain-focused pediatric critical care has emerged as an interdisciplinary model that integrates rehabilitation, circadian support, family-centered care, and developmental care as core principles across NICU, PICU, and CVICU practice

**Fig. 2:**
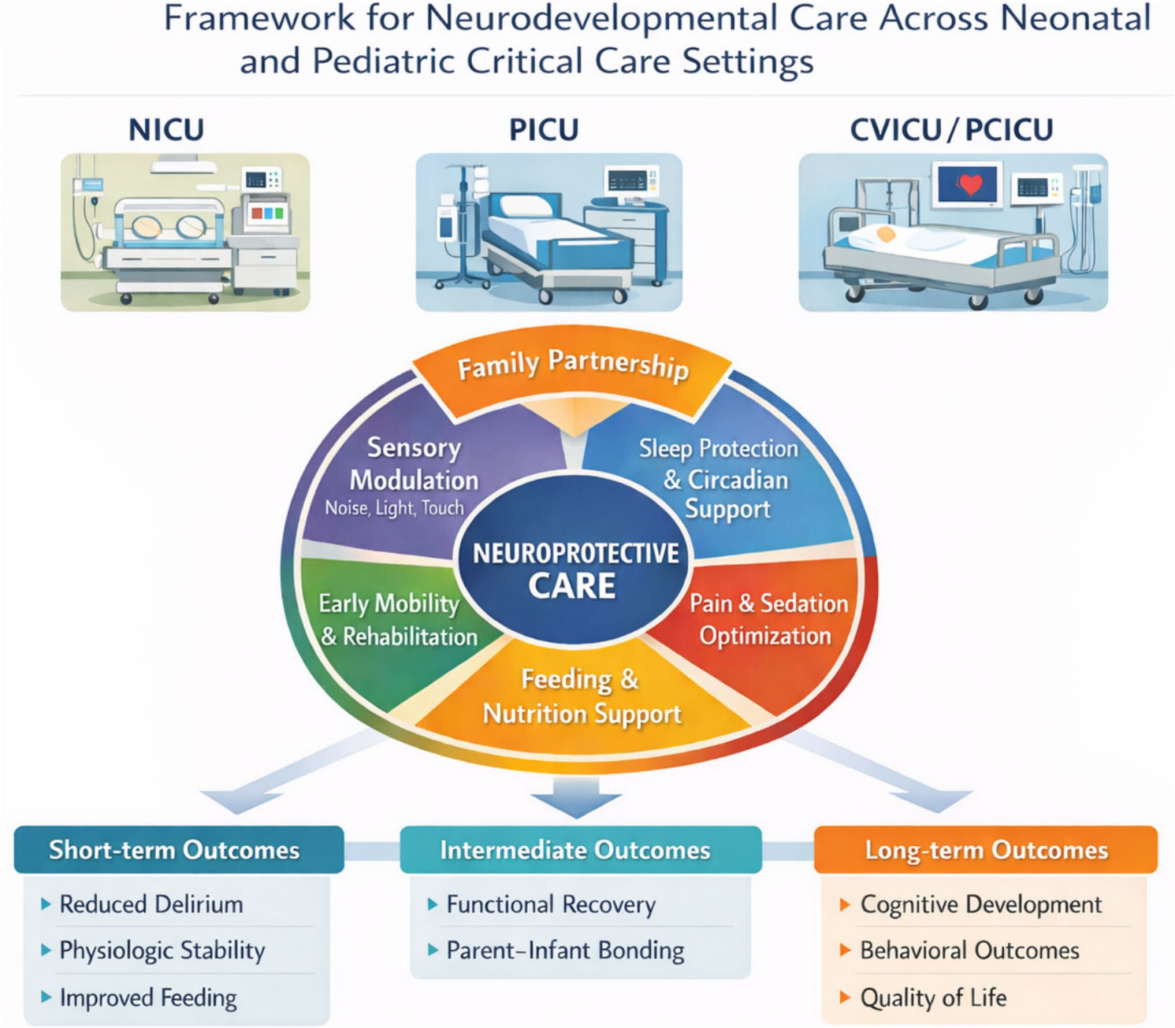
Framework for neurodevelopmental care across neonatal and pediatric critical care settings. This figure presents a cross-setting framework for neurodevelopmental care in neonatal and pediatric critical care. Neuroprotective care is positioned at the center, indicating its role as the organizing principle across unit types. Surrounding it are six interrelated domains: family partnership; sensory modulation, including noise, light, and touch; sleep protection and circadian support; pain and sedation optimization; feeding and nutrition support; and early mobility and rehabilitation. Representative images of NICU, PICU, and CVICU/PCICU settings emphasize applicability across the continuum of pediatric critical care. The framework links these care domains to a range of expected outcomes, including short-term benefits such as reduced delirium and physiologic stability, intermediate outcomes such as functional recovery and parent–infant bonding, and long-term outcomes including cognitive development, behavioral outcomes, and quality of life

Advances in neonatal and pediatric critical care have dramatically improved survival rates for high-risk infants and children. As a result, there has been a cultural shift in intensive care units—from a singular focus on survival to a broader emphasis to include neurodevelopmental outcomes and long-term quality of life. This shift originated in the neonatal intensive care unit (NICU) with approaches like the Newborn Individualized Developmental Care and Assessment Program (NIDCAP), and it now extends into pediatric (PICU) and cardiovascular intensive care units (CVICU) (Fig. 1). NIDCAP, first developed by Heidelise Als in 1984, is a comprehensive, family-centered, evidence-based model of developmental care for hospitalized newborns [1]. It introduced the philosophy that the developmental needs of the infant’s brain, such as minimizing stress, protecting sleep, and involving parents, should be integrated into every aspect of critical care. Today, this philosophy of neuroprotective, individualized care has inspired systemic changes in NICUs and is increasingly influencing care delivery in PICUs and CVICUs worldwide (Fig. 2). Below, we explore recent research, global practices, and examples illustrating how neurodevelopmental care has become central to the culture of pediatric critical care, spanning from premature infants in NICUs to infants and children in PICUs and cardiac ICUs [1].

## NIDCAP and the neurodevelopmental focus in NICUs

Over recent decades, neonatal intensive care has evolved from a predominantly technical, procedure-driven model focused on survival, through advances in thermal stability, respiratory support, infection control, and nutrition, toward an approach that also prioritizes the infant’s neurobehavioral development. By the 1990 s and early 2000 s, growing recognition of the NICU environment’s impact on brain development and behavior accelerated the adoption of structured, developmentally supportive, and individualized care for preterm and medically fragile infants [2] In NICUs, the NIDCAP approach reframed care around a relationship-based, infant-driven philosophy that recognizes each infant as an active communicator of their developmental needs. Rather than prescribing a set of universal interventions, NIDCAP uses structured behavioral observation to understand the unique self-regulatory capacities of each infant and to cocreate a caregiving plan with the family that supports the infant’s emerging competencies [3]. Trained caregivers continuously read each infant’s behavioral and physiological cues, including signs of approach, stress, and readiness, and modulate handling, sensory input, and the pacing of caregiving so that interactions are matched to what the infant can tolerate and benefit from at that moment. The goal is not simply to dampen stimuli but to support the infant’s neurobehavioral organization, protect sleep, nurture the parent–infant relationship, and ensure that the family remains the infant'’ primary regulatory partner throughout the NICU stay. The goal is to create an environment more akin to the womb or a nurturing home—promoting sleep, reducing noxious stimuli, and involving parents in caregiving. These practices help mitigate the mismatch between what a baby’s developing brain “expects” (gentle sensory input, sleep, and parental closeness) and what the hospital environment traditionally provides (bright lights, loud noise, and frequent disruptions) [4]. Over the past few decades, NICUs globally have adopted core NIDCAP principles: individualized lighting and controlling/minimizing noise, cue based procedures and routine care to allow undisturbed rest, using positioning aids to mimic the womb’s containment, and encouraging skin-to-skin contact (kangaroo care) and breastfeeding. Parents are viewed as primary caregivers and partners in the NICU, not just visitors [3, 5]. This family-integrated model reduces parental stress and fosters bonding, which in turn benefits infant brain development [5].

Critically, NIDCAP and similar developmental care programs have shown concrete benefits for premature infants. Studies have demonstrated that developmentally supportive NICU care can improve infants’ neurological development and reduce long-term developmental disorders and disabilities [6]. For example, preterm infants cared for under NIDCAP frameworks exhibit more mature brain structure and function on imaging and neurobehavioral tests compared to those receiving standard NICU care [7]. Clinical trials and meta-analyses have reported outcomes such as improved motor function, better regulation of sleep–wake cycles, and even shorter hospital stays in NICUs that implement structured developmental care [8, 9]. Although some studies have debated the magnitude of long-term impacts, many NICUs consider developmental care the standard of care because it clearly promotes healthier short-term physiology and supports later developmental milestones [9].

In short, the NICU culture worldwide has shifted to “baby-friendly” unit designs and routines, such as private or semiprivate rooms to minimize overstimulation, adjustable ambient lighting and noise-abatement measures, and staff training in reading infant cues and clustering care. This neurodevelopmental foundation established in neonatal care sets the stage for similar philosophies now permeating pediatric and cardiac intensive care units [10].

## Extending developmental care into pediatric ICUs (PICUs)

Historically, PICUs prioritized acute life support (ventilation, hemodynamic stability, and critical procedures), often with heavy sedation and restricted family presence. However, growing evidence of new morbidities in PICU survivors, including cognitive, emotional, and functional impairments, has prompted a reevaluation of PICU care practices [11, 12]. The pediatric ICU environment has likewise begun to embrace neurodevelopmental and family-centered care principles [13]. Approximately one-third of children who survive a PICU admission show neurodevelopmental impairments and reduced quality of life in the years after discharge [11]. These deficits span multiple domains (attention, executive function, social cognition, memory, and behavior) and resemble a pediatric version of post-intensive care syndrome. Notably, the behavioral and neurodevelopmental phenotypes observed in PICU survivors, including difficulties with attention regulation, executive function, sensory processing, emotional reactivity, and self-regulation, closely parallel those described in preterm infants exposed to NICU-related stressors. This convergence suggests shared mechanisms of vulnerability in the developing brain when exposed to pain, sensory overload, sleep disruption, sedation, and separation from caregivers, and provides a strong biological and clinical rationale for translating NIDCAP-informed developmental care principles into the PICU setting. In response, PICUs around the world are undergoing a cultural shift to not only save lives but also safeguard long-term development [14, 15].

Family-centered care and minimization of toxic stress are now key tenets in many PICUs. For example, most PICUs support or even encourage 24/7 parental presence at the bedside, recognizing that a parent’s comfort and voice can soothe the child and reduce anxiety. Treatment teams actively involve parents in routine care decisions and comfort measures, extending the NICU’s partnership model into the PICU setting [16, 17]. Researchers suggest an association between parental mental well-being and clinical outcomes in children with congenital heart disease; therefore, it is a clinical responsibility of the healthcare team to address and support parental psychological well-being as part of comprehensive patient care [18]. At the same time, teams should recognize that many parents cannot be at the bedside consistently, especially during daytime hours, because of distance, work, transportation barriers, or caregiving responsibilities for other children. In addition, statutory maternity, paternity, and parental leave provisions are a critical structural determinant of bedside presence. In many jurisdictions, parental leave entitlements are insufficient to cover prolonged neonatal or pediatric hospitalization, leaving parents to choose between paid employment and time with their hospitalized child. Hospitals and policymakers should recognize the impact of leave policies on family integration into ICU care, and clinicians should advocate, where possible, for extended or protected leave for parents of critically ill infants and children. We must avoid language or practices that unintentionally create guilt and instead acknowledge the significant emotional strain families experience while being pulled between home and hospital. Creative, flexible approaches can help sustain partnership when in-person presence is limited, including telehealth participation, asynchronous updates, and off-hours family conferences. Nonpharmacologic interventions that were once considered “nice-to-have” are increasingly standard: Child life specialists engage children in play or age-appropriate activities to reduce fear [19], music or art therapy is offered when possible [20], and day–night cycles are respected (dimming lights at night, cue-based cares to protect sleep) [21]. These changes address the PICU environment as a therapeutic factor itself, aiming to reduce delirium and developmental regression that can be triggered by round-the-clock noise, disorienting lighting, and isolation from normal stimuli [22].

Importantly, critical care societies have begun issuing guidelines that integrate developmental and neuroprotective goals. In 2022, the Society of Critical Care Medicine (SCCM) published the PANDEM guidelines (pain, agitation, neuromuscular blockade, delirium, environment and early mobility) for critically ill infants and children [23]. These guidelines underscore that managing pain and sedation more thoughtfully, monitoring and preventing delirium, promoting early mobility (even passive range of motion or sitting up when possible), ensuring good sleep hygiene, and actively engaging families in care are vital to improving PICU outcomes [23]. This comprehensive approach aligns with the adult ICU Liberation (ABCDEF bundle) philosophy but tailored to pediatrics. For instance, rather than automatically deeply sedating an intubated toddler, clinicians now strive for extubation as early as safely possible and in the meantime utilizing the minimum sedation necessary, frequent pain assessments, and opportunities for the child to wake, interact, and even participate in therapies each day—all while parents comfort and reassure the child if possible [24]. Such practices have been shown to reduce pediatric delirium (which affects up to 60% of PICU patients) and may prevent the cognitive decline associated with prolonged delirium [23, 25]. Indeed, delirium in ICU patients is independently linked to persistent cognitive impairment after discharge [26], so strategies that create a more normal and engaging environment in the PICU have neuroprotective value. It should be acknowledged that, while delirium screening and management are well validated in older pediatric populations, neonatal delirium remains an emerging area of evidence. Current screening tools, including the Cornell Assessment of Pediatric Delirium (CAPD), have limited validation data in the neonatal population, and ongoing work is expected to clarify both the prevalence and the optimal assessment approach for delirium in newborns.

An example of the PICU’s evolving culture is the introduction of developmental rounds or teams in some hospitals [27]. These multidisciplinary teams (including physicians, nurses, therapists, and psychologists) focus on each child’s developmental and emotional needs during daily rounds—discussing pain management, sleep quality, mobility goals, and family concerns in parallel with medical management [27]. By viewing the child holistically, PICU staff are better able to support milestones even during critical illness (for example, allowing a school-aged child to have tutoring or play time once stable or encouraging an infant to practice sitting or reaching when off the ventilator). In summary, PICUs are increasingly mirroring NICU’s neurodevelopmental principles: “All care is brain care” [28]. This additional focus is evidenced by systematic changes in care protocols, unit design (many newer PICUs feature private rooms with space for parents to sleep, noise reduction technologies, and flexible visiting policies), and staff education that emphasizes long-term outcomes alongside immediate survival [29].

## Neurodevelopmental care in the cardiac ICU (CVICU) for high-risk infants

**Fig. 3:**
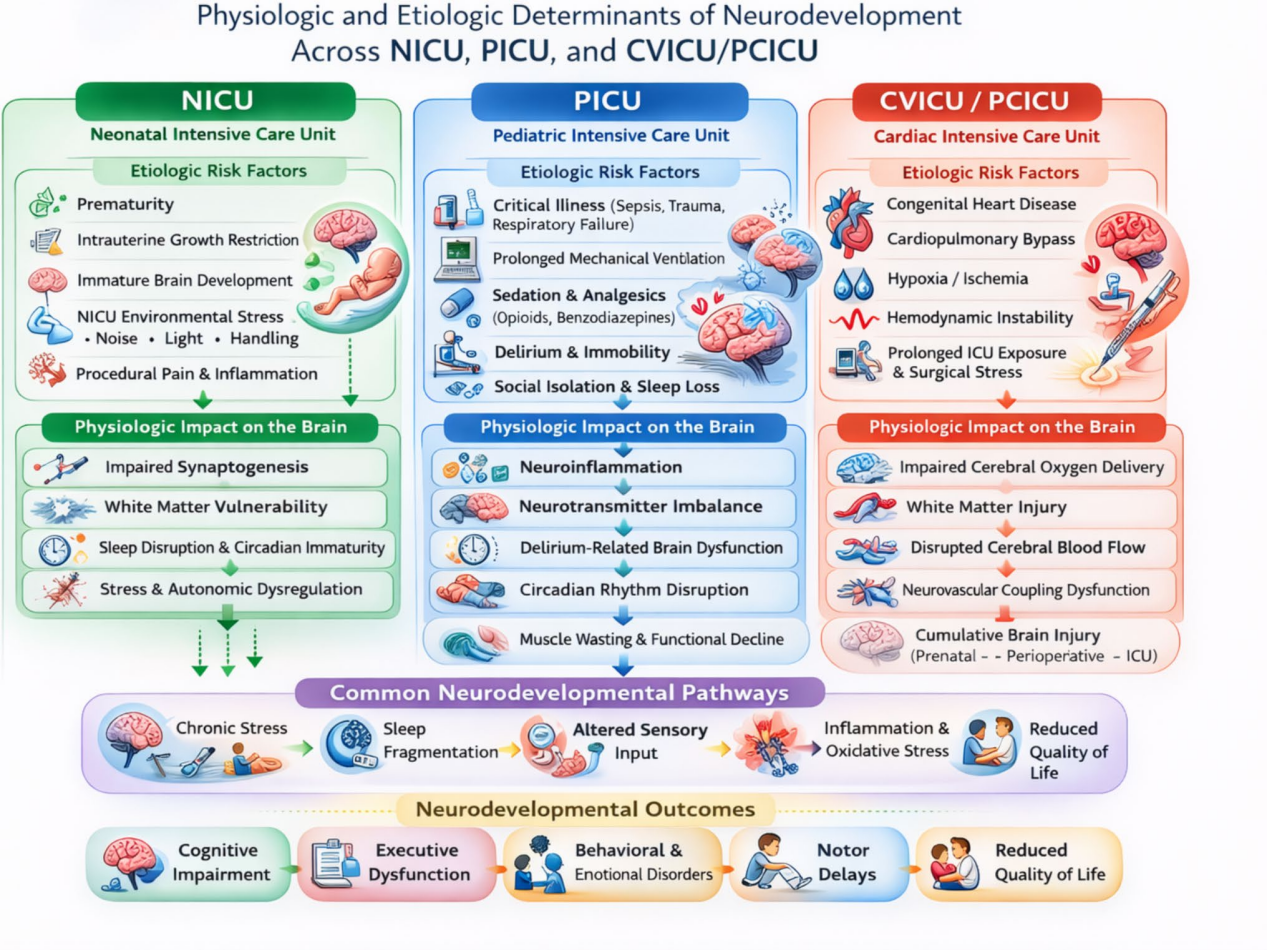
Physiologic and etiologic determinants of neurodevelopment across NICU, PICU, and CVICU/PCICU. This figure depicts the distinct but converging etiologic and physiologic pathways that influence neurodevelopment in NICU, PICU, and CVICU/PCICU settings. The NICU panel emphasizes prematurity-related and environment-related vulnerabilities, including growth restriction, immature brain development, environmental stress, and procedural pain, which are associated with impaired synaptogenesis, white matter vulnerability, sleep disruption, and autonomic dysregulation. The PICU panel highlights acquired critical illness factors, such as sepsis, trauma, respiratory failure, prolonged mechanical ventilation, sedation, delirium, immobility, and isolation, which contribute to neuroinflammation, neurotransmitter imbalance, acute brain dysfunction, circadian disruption, and functional decline. The CVICU/PCICU panel focuses on congenital and perioperative cardiac factors, including congenital heart disease, cardiopulmonary bypass, hypoxia–ischemia, hemodynamic instability, and surgical stress, which contribute to impaired cerebral oxygen delivery, white matter injury, disrupted cerebral blood flow, neurovascular dysfunction, and cumulative brain injury. Across all settings, these mechanisms converge on shared neurodevelopmental pathways that can result in cognitive impairment, executive dysfunction, behavioral and emotional disorders, motor delays, and reduced quality of life

Perhaps the most striking extension of NICU’s developmental care philosophy has occurred in the cardiac intensive care units that manage infants with complex congenital heart disease (cCHD). Pediatric cardiac surgery and postoperative intensive care emerged in the mid-twentieth century and, for decades, were guided by an almost exclusive focus on survival. Neonatal cardiac surgery developed later as diagnostic and perioperative advances enabled earlier recognition, stabilization, and intervention for cCHD. As survival improved and dedicated pediatric cardiac ICUs expanded (particularly from the 1990 s onward), neurodevelopmental morbidity in childhood became increasingly apparent during follow-up. Accordingly, early research centered on perioperative contributors to brain injury, especially cardiopulmonary bypass and the operative course, before broadening to the circulatory effects in the fetus as well as the cumulative effects of critical illness and prolonged hospitalization (Fig. 3). In contrast to NICUs, systematic neurodevelopmental care in CICUs gained real momentum only in the 2010 s, reflecting a shift from “survival” to survivorship that prioritizes long‑term function and quality of life [30]. Children with cCHD (such as those who require open-heart surgery in the neonatal period) have emerged as the highest-risk cardiac population for neurodevelopmental disorders, despite often being born at full term. Research over the past decade has revealed that developmental delays and disabilities are the most common morbidity in survivors of complex CHD, exceeding even the rates of physical complications like reoperations or arrhythmias [31, 32]. These children frequently face challenges in cognition, language, attention, executive function, motor skills, social cognition, and behavior that can persist into adolescence [31, 33]. The neurodevelopmental impairments in many cCHD survivors translate into lower academic achievement, greater need for special education services, and diminished long-term quality of life [31, 34–36]. Fetal brain imaging studies show that many infants with critical CHD have abnormal fetal brain development characterized by white matter injury or delayed brain maturation, which is then compounded by the stresses of intensive care (pain, bypass surgery, and prolonged hospitalization) in the newborn period [37–40]. Recognizing this, clinicians and researchers have argued that the CVICU environment and care practices should be modified to protect the brain, just as NICUs have done for preterm infants.

**Fig. 4:**
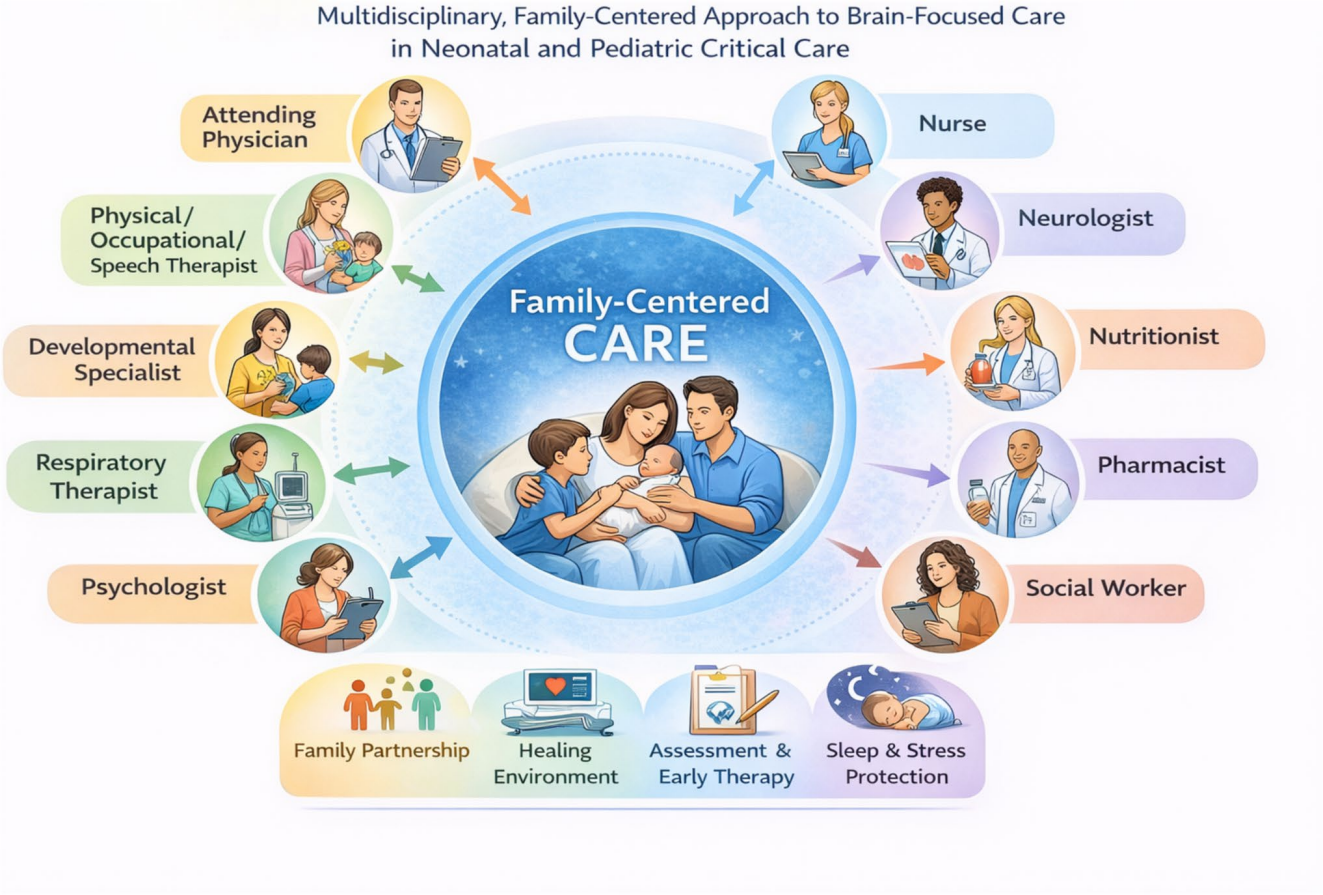
Multidisciplinary, family-centered approach to brain-focused care in neonatal and pediatric critical care. This figure illustrates a multidisciplinary, family-centered model for brain-focused care in neonatal and pediatric critical care. Family-centered care is positioned at the center, underscoring the role of the child and family as the core of all care planning and delivery. Surrounding the family is an interdisciplinary team that includes an attending physician, nurse, neurologist, nutritionist, pharmacist, social worker, psychologist, respiratory therapist, developmental specialist, and physical, occupational, and speech therapists. The circular arrangement conveys coordinated, shared responsibility around the family to advance brain protection and developmentally supportive care. The foundational elements shown below the central image—family partnership, healing environment, assessment and early therapy, and sleep and stress protection—represent core domains through which the multidisciplinary team operationalizes neuroprotective critical care

A landmark cultural change has been the deliberate introduction of NIDCAP principles into the cardiac ICU. Over the past decade, this shift has been reflected in the expansion of formal, interdisciplinary neurodevelopmental programs within cardiac intensive care units, designed to proactively mitigate neurologic risk during hospitalization rather than addressing neurodevelopmental sequelae only after discharge. These programs integrate neurologists, developmental specialists, nurses, and rehabilitation specialists into routine cardiac critical care, signaling a broader cultural move toward structured neurocardiac care models [41]. Some also include pharmacists and nutritionists on a routine basis (Fig. 4).

Several pediatric cardiac ICUs now offer concrete, reproducible models for embedding neurodevelopmental care into high-acuity CVICU/PCICU practice. At Boston Children’s Hospital, a team led by developmental psychologists and cardiac ICU nurses launched an initiative to implement individualized developmental care for infants with CHD, beginning with staff training in NIDCAP concepts and tailoring care to a similarly “brain-critical” period as the NICU [42, 43]. In Denver, Children’s Hospital Colorado established the Cardiac Inpatient Neurodevelopmental Care Optimization (CINCO) program, an interdisciplinary, low-cost bundle (developmental kits, EHR order panels, bedside developmental plans, caregiver mental health supports, and developmental care rounds) with growing implementation over time and associations with fewer delirium days and higher BSID-4 cognitive scores after implementation [44, 45]. In Washington, DC, Children’s National Hospital described building and sustaining an inpatient Neurocardiac Critical Care Program in a 26-bed pediatric cardiac ICU, anchored by consistent neurologist engagement, comprehensive education, evidence-based practice changes, and coordinated QI/research aimed at proactively mitigating risks to the developing brain and family unit [41].

What makes the cardiac population unique is that these infants are often full-term and acutely post-surgery, yet their brains still “expect” gentle, patterned sensory input and parent closeness for optimal development [46]. In practice, bringing NIDCAP into the CVICU has meant altering both caregiving and the unit environment to better meet infant needs. Lighting is individualized and sound is reduced whenever safely possible, even in a technology-dense cardiac unit. Care is planned and paced according to each infant’s tolerance—nurses learn to read subtle cues of stress or stability and time interventions (like suctioning, exams, or repositioning) to avoid overstimulation [46]. Perhaps most notably, the CVICU has worked to increase family involvement: Parents are encouraged and assisted to hold their infants, even those with chest tubes, central venous catheters and/or ventilators, as soon as it is medically feasible. A “holding protocol” was developed so that nearly every cardiac infant can be held by a caregiver daily, given the evidence that skin-to-skin contact stabilizes infant physiology and even improves cognitive and autonomic function in this population [47, 48]. Additional developmental supports include engaging physical and occupational therapists early for bedside motor exercises (for example, “tummy time” when the infant is awake and medically stable) to promote motor development despite the constraints of surgery recovery. Early studies have found that even infants who have undergone open-heart surgery can safely participate in prone positioning and therapy, resulting in better motor outcomes by hospital discharge [49].

Implementing these changes in a CVICU setting has required a significant systemic and cultural shift [46]. Cardiac ICU staff, from nurses and doctors to respiratory therapists and surgeons, traditionally focused on hemodynamics and postoperative care, often in a fast-paced, stimulation-rich environment. Now, they are being retrained to incorporate a developmental lens into their practice as well. Boston Children’s, for example, created an interdisciplinary “team of champions” (including bedside nurses, nurse practitioners, therapists, and child life specialists) who underwent focused training in developmental care and then spread these practices unit-wide [42]. Leadership support was secured early on to ensure buy-in; nurse managers, cardiac surgeons, and cardiac intensivists were brought into the discussion so that developmental care became a shared priority rather than an “optional” add-on [50]. Over time, this has led to myriad small but impactful changes: adjusting monitor alarm volumes [51], establishing quiet hours, shielding infants’ eyes from unnecessary light, and designing bedside schedules that prioritize protected sleep [52]. In some cases, physical renovations or equipment changes are pursued—for example, adapting multipatient cardiac units or older PICUs to include more private spaces or dimmable lighting for infant patients [53]. As one cardiac ICU nurse described, “*although we’ve been practicing some pieces of NIDCAP all along, implementing the entire NIDCAP approach is a huge cultural shift*,” and it requires continuous staff education and adaptation [50]. It cannot be emphasized enough that these programs must be actively supported by cardiac surgeons and intensivists. Early data and anecdotal reports are encouraging: Clinicians believe these modifications will lead to better long-term developmental trajectories and quality of life for children with CHD, complementing the lifesaving cardiac interventions they receive [42]. The American Heart Association’s recent science advisory on developmental care in CHD amplifies this message, calling for hospital infrastructure and research to make neurodevelopmental care a standard part of cardiac critical care in the coming decade [49].

## Key components and strategies of neuroprotective care

Neuroprotective and developmental care in the NICU, PICU, and CVICU shares a common aim: to preserve physiologic stability and surgical recovery while actively supporting brain development during a period of heightened vulnerability. Although workflows and patient populations differ across units, a consistent set of domains underpins effective practice (Table 1). These domains translate neurodevelopmental principles into bedside routines and system-level infrastructure that can be implemented without compromising safety. At the same time, it is important to acknowledge that full NIDCAP implementation, which requires extensive trainer-led education, ongoing observation, and substantial institutional resources, may not be feasible across all units, particularly in low-resource settings, smaller centers, or nonacademic hospitals. Reassuringly, emerging evidence suggests that even targeted developmental care education delivered to bedside staff in surgical and high-acuity neonatal settings can yield meaningful benefits: reductions in infant stress responses (e.g., improved heart rate variability, fewer behavioral stress cues, and shorter periods of inconsolability) and, for parents, improved perceptions of emotional support at discharge, a factor with potential downstream implications for infant and family mental health [54]. This evidence supports a graduated, equity-informed implementation strategy: Units that cannot pursue full NIDCAP certification can still deliver meaningful neuroprotective care by investing in structured developmental care education, interdisciplinary champions, and family-integrated practices appropriate to their resource context.

**Table 1:** Core domains and strategies of neuroprotective and developmental care across NICU, PICU, and CVICU

Domain	Rationale	Key bedside strategies	Implementation notes (NICU/PICU/CVICU)	Indicative outcomes/metrics
Family involvement and empowerment^#^	Parent partnership supports coregulation, emotional security, and caregiver resilience [13, 16, 55, 57–59]	Open visitation; parent participation in routine caregiving (feeding/bathing/comforting); shared decision-making; parent education/support groups [13, 16, 55]	NICU: normalize bedside presence + cue education [13, 16, 55] PICU: structured psychosocial support to reduce caregiver distress [57, 58] CVICU: staged engagement models (e.g., Care Partnership Pyramid), acknowledging mixed early outcome signals [56]	Parent presence hours/day; % family participation in rounds; caregiver depression/anxiety/PTSS screening uptake [57–59]
Cue-based, individualized care	Tailoring care to behavioral/physiologic tolerance reduces overstimulation and supports self-regulation and sleep-dependent neurodevelopment [60–68]	Observe cues (vitals/color/state regulation); pause/modify care when stressed; plan care around readiness; cluster care to protect sleep–wake cycles [60–64]	NICU: cue-based handling with stress-sign monitoring [60–62] PICU: align interventions to age-appropriate rest/interaction windows [63, 64] CVICU: pacing care to minimize post-op fatigue while protecting sleep [63, 64]	Protected sleep blocks; proportion of clustered-care adherence; fewer stress-related instability events [63–67]
Optimizing the ICU environment^†^	ICU sensory overload (noise/light/touch) can affect neurobehavior; circadian support and pain/stress reduction are neuroprotective [23, 24, 47, 69–76]	Noise reduction (alarm stewardship/quiet voices/ear protection); cycled lighting/dimming at night; quiet hours; positive touch and skin-to-skin; multimodal pain control including nonpharmacologic comfort measures [23, 24, 47, 69–76]	NICU: established sound/light standards + shielding [72] PICU/CVICU: adapt NICU sensory standards to tech-dense environments; emphasize circadian orientation [72, 74]	Unit noise/light audits; quiet-hour adherence; pain scores/analgesia timing; delirium screening (PICU and CICU) [23, 24, 73, 74]
Supportive positioning and motor stimulation/early mobility	Prevents deformity, weakness, and developmental delays; mobility programs link to functional recovery and delirium reduction [77–80]	Nesting/swaddling for midline flexion; safe handling; ROM; tummy time when feasible; progressive mobility (sit/transfer/ambulate) [77–79]	NICU: positioning to support self-regulation and musculoskeletal development [77, 78] PICU: structured early mobility protocols [79] CVICU: therapist-guided, safety-aware activity even post sternotomy, with evidence of tolerance and motor benefits [80]	Mobility level/day; PT/OT consult timing; delirium days; functional status at discharge [79, 80]
Nutrition and feeding as neurodevelopmental therapy	Feeding integrates sensory-motor learning, growth, and bonding; targeted support reduces aversion and improves neurodevelopmental trajectory [81–87]	Cue-based feeding; prefeeding interventions including skin-to-skin contact, nonnutritive sucking, scent-pad exchange, and oral care with expressed breast milk; protect oral experiences; speech/OT support; promote maternal milk and donor milk when needed; involve parents in feeding skills [81, 83–85]	NICU: cue-based feeding + nonnutritive sucking strategies [81] CVICU: anticipate fatigue/vocal cord issues and post-op barriers (e.g., baseline tachypnea secondary to sternotomy, particularly in neonates, diaphragm paresis); early therapist involvement [82, 83] PICU: stepwise feeding rehabilitation after critical illness [83]	Time to full PO feeds; % feeds by parents; growth velocity; oral aversion rates; caregiver confidence/stress measures [82, 83, 86, 87]
Staff training and interdisciplinary collaboration	Consistency requires shared mental models and workflow integration of developmental goals [88–90]	Training (e.g., NIDCAP); interdisciplinary developmental plans; champions/rounds; checklists/flowsheets documenting developmental actions [88–90]	All settings: embed developmental care in daily routines and documentation to prevent optional implementation [90]	% staff trained; documentation completion rates; adherence to developmental care bundle elements [88–90]
Continuity of care beyond the ICU	Developmental risk persists throughout childhood. post-discharge; surveillance and early intervention reinforce ICU gains across childhood [32, 91–96]	Follow-up clinics; early-intervention referral pathways; longitudinal neurodevelopmental monitoring and feedback loops [32, 91, 92]	NICU: established NICU follow-up clinics [91] CVICU: cardiac neurodevelopmental programs through school age [32] PICU: emerging post-ICU survivorship/post-ICU clinics for cognitive/psychologic sequelae [92–94]	Follow-up attendance; referral completion; developmental screening rates; long-term outcomes registry capture [32, 91–94]

Table summarizing seven domains of neuroprotective care (family partnership, cue-based care, ICU environment, positioning/mobility, nutrition/feeding, staff training/teamwork, and continuity after discharge), with rationale, bedside actions, setting-specific implementation notes (NICU/PICU/CVICU), and example metrics/outcomes

Abbreviations: *NICU* neonatal intensive care unit, *PICU* pediatric intensive care unit, *CVICU* cardiac intensive care unit, *ROM* range of motion, *PO* per os (oral feeding), *PT/OT* physical/occupational therapy, *PTSS* post-traumatic stress symptoms

^#^Family bedside presence (including timing and duration) is shaped by home responsibilities, distance, fear, statutory maternity/paternity/parental leave entitlements, and, in neonates, postpartum recovery, so it should be measured thoughtfully without treating “more presence” as a simple marker of success

^†^Delirium screening in neonates remains an emerging area of evidence; current tools have limited validation in this population

*Family involvement and empowerment* are foundational, positioning caregivers as partners in daily care and decision-making. Family-centered policies and structured education (e.g., recognizing infant cues, participating in routine caregiving) promote parental confidence and reduce distress, which may otherwise disrupt bonding and coregulation [13, 16, 55, 57, 58]. In high-acuity cardiac settings, staged engagement frameworks such as the Care Partnership Pyramid can help calibrate parental roles to illness severity and clinical stability; early evaluations have not consistently demonstrated measurable short-term clinical differences, but the approach remains valuable for guiding participation and supporting caregiver experience [56–59].

*Cue-based individualized care* replaces rigid routines with tailored pacing guided by behavioral and physiologic cues. In neonates, this includes monitoring state regulation and signs of overstimulation (e.g., autonomic changes) and modifying care accordingly. In older children it may involve aligning interventions with readiness for interaction and protected rest [60–62]. Individualizing care and deliberate protection of sleep/wake cycles are emphasized because sleep supports both surgical recovery and neurodevelopment and may mitigate the cumulative impact of repeated stressors [63, 64]. By adjusting timing and intensity of care to tolerance, teams can attenuate stress responses that, when persistent, may adversely affect the developing brain [65–67]. Such individualized approaches have been associated with improved neurodevelopmental outcomes in preterm populations and provide a rationale for extension to broader pediatric critical care contexts [68].

*A neuro-friendly CVICU environment* addresses sensory overload and circadian disruption common to critical care. Strategies include noise reduction (e.g., alarm stewardship), cycled lighting and day/night orientation, and scheduled quiet periods [69–74]. We should also challenge convenience-based routines that fragment sleep, such as waking children at 5 AM for chest radiographs or nonurgent tasks timed primarily for team workflow. Whenever clinically safe, teams should deliberately bundle care (e.g., labs, imaging, echocardiography, assessments, and nursing interventions) and align timing with the child’s sleep–wake cycles to reduce repeated disruptions, support recovery, and preserve a more developmentally protective environment. Positive tactile input, such as gentle touch and skin-to-skin care, can provide supportive sensory experiences, while minimizing unnecessary noxious stimuli [47, 75]. Pain is treated as a neurodevelopmental stressor; thus, pharmacologic analgesia is paired with nonpharmacologic comfort measures to reduce physiologic and behavioral consequences of unmanaged pain [23, 24, 76].

*Supportive positioning, motor stimulation, and early mobility* aim to prevent weakness, deformity, and developmental delay associated with prolonged immobility [77, 78]. Progressive mobility, from range-of-motion activities to sitting, transfers, and ambulation, is introduced as soon as medically feasible; pediatric mobilization programs have been linked to reduced delirium and improved functional recovery [79]. In CVICU settings, post-sternotomy infants generally tolerate appropriately modified therapy well, with reports of improved motor skill trajectories compared with prolonged bed rest [80].

*Nutrition and feeding* are approached as neurodevelopmental therapies. Cue-based feeding supports sensory-motor learning and bonding, while speech/occupational therapy involvement can prevent oral aversion, particularly relevant for cCHD infants facing fatigue or vocal cord injury risk [81–83]. Importantly, oral feeding readiness is built well before the first oral feed. Prefeeding interventions, including skin-to-skin contact, nonnutritive sucking on a pacifier or at the emptied breast, scent-pad (parental scent cloth) exchange, and oral care with expressed breast milk, provide positive, parent-mediated oral and olfactory experiences that are associated with reduced stress, improved physiologic stability, earlier transition to oral feeds, and better long-term feeding outcomes. Human milk is prioritized where possible, with donor milk strategies when maternal milk is unavailable, given associations with favorable neurodevelopmental outcomes [84, 85]. Effective feeding practices support growth and may influence later cognitive and sensory outcomes while also reducing parental stress and enhancing confidence [86, 87].

Finally, sustainable implementation depends on *staff training and interdisciplinary collaboration*, including structured documentation to make developmental goals visible and routine [88–90]. Neuroprotective care also requires *continuity beyond the ICU*, including on the general ward, outpatient follow-up clinics, and early intervention pathways, well established in NICU programs and increasingly formalized in cardiac neurodevelopmental and post-PICU survivorship models [32, 91–96].

## Outcomes and evidence of impact

While the compassionate logic of developmental care is clear, it is backed by a growing body of evidence demonstrating its benefits [5, 6, 97]. In neonatal populations, decades of research have linked neuroprotective care to improved short-term and long-term outcomes [97]. Preterm infants who received structured developmental care (e.g., in NIDCAP trial units) showed more optimal brain maturation on MRI and better neurodevelopmental scores in infancy, including improvements in motor skills, attention, and self-regulation, compared to controls [97]. Some studies have noted reductions in medical complications as well, such as shorter durations of respiratory support and shorter hospital stays with comprehensive developmental care programs [98]. Although earlier meta-analyses called for larger trials, the consensus in practice is that the NICU developmental care improves the overall developmental trajectory and medical stability of vulnerable infants [43, 98]. This is evident in the near-universal adoption of at least some form of developmental care in modern NICUs around the globe [99].

For PICU survivors, the move toward neurodevelopmental care is more recent, but early data are starting to underscore its importance. As mentioned, about one in three PICU survivors may experience neurodevelopmental impairment, ranging from mild learning difficulties to severe cognitive or motor deficits [100]. These impairments are often accompanied by behavioral or emotional challenges (such as ADHD-like symptoms or anxiety) and can significantly reduce quality of life [101, 102]. By addressing factors like delirium, pain, and immobility in the PICU, we aim to prevent adding insult to an already stressed developing brain [21, 23–25]. Programs that implemented bundled approaches (pain prevention, minimal necessary sedation, delirium monitoring, mobilization, and family engagement) have demonstrated tangible improvements, including fewer days of delirium and a reduction in ICU length of stay, which correlate with better cognitive outcomes at discharge [21, 23–25]. It is intuitive and increasingly evidenced that a child who spends less time delirious or deeply sedated in the ICU will have less disruption to memory and thinking processes, translating to smoother cognitive recovery [103]. Moreover, qualitative outcomes like preserved developmental milestones are reported: For example, toddlers in a PICU that emphasizes play and mobility may continue to gain language and motor skills during their hospitalization, rather than regressing or stagnating [104].

In the cardiac ICU context, formal outcomes research is underway, propelled by statements from organizations like the AHA calling for rigorous evaluation of developmental care interventions [49]. Early results and multicenter studies point to several positive trends. Neurodevelopmentally focused cardiac care has been associated with improved feeding outcomes and growth, which are critical for brain development in infants with CHD [67, 105, 106]. Infants who experience more skin-to-skin holding in CVICU not only show calmer behavior and more stable vital signs, but in one study, they later had better cognitive scores and neurobehavioral regulation compared to those who had minimal holding [47, 107]. Reducing ambient stress in CVICU (for instance, through private rooms and noise control) has been linked to lower levels of physiological stress markers in infants, suggesting a more supportive milieu for brain development [72, 74, 75, 108]. On the family side, parents involved in developmental care initiatives report lower anxiety and stronger attachment to their infants, which is likely to benefit the child’s emotional development long after ICU discharge [49, 58, 59]. Looking further ahead, the collective goal is to see fewer school-age children with CHD needing special education or therapies and decreasing numbers of families suffering from PTSD, because we intervened early in the ICU [109]. Indeed, by mitigating early neurodevelopmental injury, we hope to alter life-course outcomes—enabling survivors of NICU, PICU, or CVICU to enter childhood with healthier cognitive and emotional skills. Longitudinal studies are being designed to track such children into school years and adolescence [110]. Initial evidence is promising, but even more compelling is the universal improvement in humanistic outcomes: Infants in developmental ICUs spend more time in calm states, are easier to console, and have parents who feel empowered and bonded [42, 111]. These are outcomes that, while hard to quantify, speak to a better quality of early life experience—something that in itself justifies the cultural paradigm transformation of our ICUs with sustainable focused programs [41].

## Conclusion

The integration of neurodevelopmental care into NICU, PICU, and CVICU settings represents a fundamental cultural shift in pediatric critical care. No longer are intensive care units solely judged on survival rates; they are now also measured by how well they support the growing brain and the family unit during and after critical illness. This shift, pioneered by programs like NIDCAP in the NICU, has led to tangible changes in unit design, care delivery, and staff training across the continuum of care—from the premature neonate fighting to grow, to the critically ill toddler or the infant recovering from heart surgery. Hospitals around the world have embraced elements like family-centered rounds, quiet healing environments, and early rehabilitative interventions, reflecting a consensus that “survival is not enough; our children deserve to thrive.”

The journey is ongoing. Continued research and quality improvement efforts are needed to fine-tune developmental care practices and demonstrate their long-term benefits on cognitive development, emotional well-being, and life achievements. Nevertheless, the existing evidence and experience strongly support this holistic approach. Neuroprotective, developmental care strategies not only improve clinical outcomes in the short term but also honor the dignity and potential of each child and family in crisis. In essence, the philosophy that began in the NICU has come of age: It now permeates pediatric critical care, creating a continuum of care in which the child’s neurodevelopmental future is as valued as their immediate survival. This cultural evolution, driven by both science and compassion, is reshaping intensive care units into places where healing the brain and nurturing development are woven into the fabric of saving lives.

## Data Availability

No datasets were generated or analysed during the current study.

## Abbreviations

ABCDEF: ICU Liberation bundle (assess/prevent/manage pain; both SAT/SBT; choice of sedation; delirium; early mobility; family engagement)
ADHD: Attention-deficit/hyperactivity disorder
AHA: American Heart Association
BSID-4: Bayley Scales of Infant and Toddler Development, Fourth Edition
CHD: Congenital heart disease
cCHD: Critically congenital heart disease
CINCO: Cardiac Inpatient Neurodevelopmental Care Optimization
CVICU: Cardiovascular intensive care unit
EHR: Electronic health record
ICU: Intensive care unit
NICU: Neonatal intensive care unit
NIDCAP: Newborn Individualized Developmental Care and Assessment Program
PANDEM: Pain, agitation, neuromuscular blockade, delirium, environment and early mobility
PCICU: Pediatric cardiac intensive care unit
PICU: Pediatric intensive care unit
QI: Quality improvement
SCCM: Society of Critical Care Medicine

## Declarations

### Use of gen-AI tools

Figures 1, 2, 3, and 4 were prepared with the assistance of a safe, university-based, generative artificial intelligence (ChatGPT-4o image generation) for the initial visual draft. All conceptual content, scientific framing, alt-text, and final layouts were designed, reviewed, and edited by the authors, who take full responsibility for the accuracy and appropriateness of all visual content. No identifiable patient images were used or generated.
